# Are psychiatrists only fools and horses to be open all hours?

**DOI:** 10.1192/pb.bp.114.050070

**Published:** 2015-12

**Authors:** Gary Wannan, M. E. Jan Wise

**Affiliations:** 1 Central and North West London NHS Foundation Trust, London, UK

## Abstract

The UK government's proposal for 24-hour healthcare means effectively asking doctors to work more unsociable hours for relatively little financial gain. In our opinion, psychiatry is particularly vulnerable to deterioration owing to negotiations of the terms of the current Consultant Contract that ensures fewer antisocial hours, whereas without parallel appropriate internal team and intra-agency working, provisions for which are not included in the government's proposals to extend care, patient care is vulnerable. Clarification and a narrower redefinition of what constitutes a psychiatric emergency is called for.

Psychiatry is no stranger to major shifts in the way it thinks or is delivered. The pre-pharmacological treatments of the 1950s shifted into the Brave New World of Largactil, and reacted with the therapy-rich and anti-establishment fad made famous by Laing and the railing against biological facets exemplified by Szasz. With its diversity of self-reflection, psychiatrists have a legacy to assist in changing practice.

Methods or modes of delivery of care have also been criticised. Deinstitutionalisation was followed by the crisis of care in the community, amid a spate of high-profile homicides. Risk management, best represented by the Confidential Enquiry into Suicides and Homicides^1^ and at worst an ill-thought-out bureaucratic bungle, added to the administrative burden without clear evidence that the public and patients are now safer.

Recent assaults on the financial health of medical staff working in the National Health Service (NHS) have included 6 years of pay cuts and raids on future pensions. More general cuts in the organisation they work in have led to low morale and allegations that rather than this being viewed as a new paternalism, that somehow ‘nanny [the State] knows best’, it is a neglecting and neglectful state.^2^ After several years of the ‘Nicholson challenge’,^3^ with further cuts in funding to mental health services, resources are at a dangerously low level^4^ and the Cinderella of the NHS faces unprecedented challenges, singled out for selective cuts with resources not following morbidity.

In this environment, the government is proposing a ‘24/7 service’. On the face of it, running a psychiatric service is a 24-h activity anyway. In-patients require constant access to appropriate medical and nursing help; relapses and remissions of community patients do not always coincide with their care coordinator's availability. So it seems self-evident that psychiatric services run 24 hours a day, 365 days a year. To many it seems that a disingenuous impression is being created that consultants do not provide a 24-h service when in fact they do so across specialties in the NHS.^5^ The government appears to have used this sleight to demand that there be contractual changes to remove any obligation to pay consultants an appropriate premium for out-of-hours work.

However, if the government's drive is for the same level of service all the time, that is a separate matter. Examples of this would include routine out-patient clinics on a Sunday afternoon and late evening consultant-led ward rounds. Nearly all disciplines, especially psychiatry, deliver care in a team manner, so without expansion of numbers across professions, how are the same levels of interactivity to be maintained outside Monday to Friday, 9-to-5 opening hours? If Donne's metaphor is apt, psychiatry is not an island, so where will children and families' Social Services be, and the police, occupational therapy, clinical psychology and investigative services, when required? Although the government has acknowledged this problem,^6^ it is evident that an initiative from the Department of Health will not be prioritised in the same way by other departments.

The financial and human resource implications appear to be largely not considered. Although there is evidence of better outcomes for patients seen during the week in non-psychiatric specialties, there is no clear costing of the changes required to raise weekend services to the same level as those provided during the week. Many NHS staff have premium rates for unsocial hours: fewer staff will be spread more thinly across weekends and evenings, so without additional recruitment, daytime services will shrink and likely worsen outcomes Monday to Friday. Whereas there are theoretical savings in physical medicine with extended use of surgical theatres or radiological facilities, such potential is not so easy to find in mental health services. For those drawn to community work in particular, reducing family time, a reason many choose the specialty in the first place, will affect recruitment and retention. Surveys of consultants show that the average time spent on NHS activity is already well in excess of contractual requirements, at 5.8 hours extra unpaid labour a week,^7^ thereby further increasing the challenge of doing more with less.

We are aware that that several NHS trusts are attempting to alter Standard Operating Procedure to specify what consultants will do while on call, including mandating non-emergency tasks out-of-hours. Consultants, in particular, have the reasonable expectation that on-call work will be for tasks that require their level of experience and skill, rather than for bureaucratic reasons.

The 2003 Consultant Contract^8^ contains an important provision at Schedule 3, Paragraph 6:

‘Non-emergency work after 7 pm and before 7 am during weekdays or at weekends will only be scheduled by mutual agreement between the consultant and his or her clinical manager. Consultants will have the right to refuse non-emergency work at such times. Should they do so there will be no detriment in relation to pay progression or any other matter.’

To the government, this is a major impediment to an imposition of 24/7 working in the absence of agreement. To negotiators, it is an important safeguard against excessive and antisocial hours with the compromise of patient safety. For either side, its meaning could become totemic.

There may be much discussion over what an emergency is. We suggest a framework where the most important categories are akin to those used in time management:^9^

- emergency
- urgent
- important
- not urgent not important.

Emergency is defined as ‘a serious, unexpected, and often dangerous situation requiring immediate action’. So, a non-emergency is anything that is not an ‘emergency’. An urgent problem may later become an emergency, but that is a separate issue.

The Cassandras among us may worry about litigation where an action is delayed. However, it is the commissioned service's responsibility to ensure that there are appropriate care arrangements in place, not the individual consultant's. Of course, the General Medical Council provides help on this matter when stating that:

‘If patients are at risk because of inadequate premises, equipment or other resources, policies or systems, you should put the matter right if that is possible. You must raise your concern in line with our guidance and your workplace policy.’^10:para.25^

Many will agree that acute suicidal ideation, an acute dystonic reaction and attempted self-harm are all emergencies. Likewise, while administrative procedures that allow leave while detained, or medication to be administered lawfully, are very important, they are not ‘emergency’ situations. Military training includes the mantra that ‘A failure to plan on your part does not constitute an emergency on mine’, something we would do well to remember.

So where do practitioners sit on the urgent category? There will be some that lie outside our comfort zone, for humanitarian or other reasons, such as advice to a trainee about medication for psychotic phenomena where few would not readily offer advice even when not deemed an emergency. However, where is the boundary with the Human Rights Act? Many NHS trusts have a seclusion review policy where after 24 h in seclusion a review in person by a consultant must occur. Now this may be good practice according to the Mental Health Act Code of Practice, but an infringement of best practice is not an emergency.

It may well be that the solutions of other countries may help. Greenland, part of the Kingdom of Denmark, runs practically all of its psychiatric services over videoconferencing facilities.^11^ In Los Angeles, time constraints and skills shortages have led to a parallel approach (R. Mendoza, personal communication, 2015). The British ‘special hospitals’ have used the same technology to reduce loss of clinical time to travel to disparate sites or courts.

The discussion about what is an emergency should rage, and it should rage hard. It is a point around which consensus would allow us to protect that which we cherish – a service delivered in a considered and coordinated manner, where there is a fair exchange of time and labour for salary and security. In a politically driven, evidence-poor initiative, psychiatrists should stand firm on whether the proposed changes hold merit. If they do, at what cost should we accept them?
